# Physiological and cellular requirements for successful elongation of the preimplantation conceptus and the implications for fertility in lactating dairy cows

**DOI:** 10.21451/1984-3143-AR2018-0028

**Published:** 2018-08-03

**Authors:** Eduardo de Souza Ribeiro, José Felipe Warmling Spricigo, Murilo Romulo Carvalho, Elvis Ticiani

**Affiliations:** Department of Animal Biosciences, University of Guelph, Guelph, ON, N1G 2W1, Canada

**Keywords:** conceptus elongation, dairy cow, pregnancy loss.

## Abstract

Elongation of the preimplantation conceptus is a prerequisite for maternal recognition of pregnancy and implantation in ruminants. Failures in this phase of development likely contribute for the subfertility of lactating dairy cows. This review will discuss our current understanding of the physiological and cellular requirements for successful elongation of the preimplantation conceptus and their potential deficiency in subfertile lactating dairy cows. Major requirements include the priming of the endometrium by ovarian steroids, reprogramming of trophectoderm cells at the onset of elongation, and intensification of the crosstalk between elongating conceptus and endometrium. Conceptus elongation and survival in dairy cows does not seem to be affected by lactation per se but seem to be altered in subgroups of cows with endocrine, metabolic and nutritional imbalances or deficiencies. These subgroups of cows include those suffering diseases postpartum, anovular cows enrolled in synchronization programs, and cows with low concentration of circulating steroids and IGF1. Success of conceptus elongation starts long before breeding and entails optimization of health and nutrition programs, especially during the transition period, and might be extended to the supplementation of endocrine and nutritional shortages at the time of breeding. Genetic selection will eventually become more important as researchers unravel the molecular control of reproduction and develop new fertility traits focused on pregnancy survival.

## Introduction

**Figure 1:**
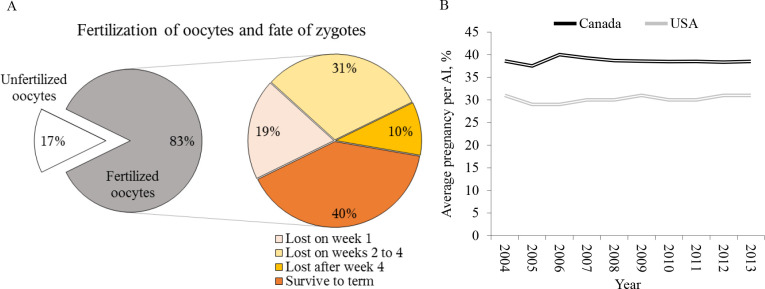
Pregnancy failures and average conception risk in lactating cows in North America. (A) Percentage of fertilize and unfertilized oocytes and fate of zygotes. (B) Average conception risk of DHI herds in Ontario and Western Canada and average conception risk of US Holstein cows in DHI herds. Data from Ribeiro (2018).

Early pregnancy losses are highly prevalent in lactating cows and lessen production efficiency in dairy herds (Ribeiro *et al*., 2012). Approximately half of the zygotes fail to survive the first 4 weeks of development, contributing meaningfully for the low average conception risk of lactating cows (Santos *et al*., 2004; Wiltbank *et al*., 2016; Ribeiro, 2018; Fig. 1). Ultimately, these losses are caused by impaired developmental competence of the zygote and/or inadequate uterine environment, which in turn are influenced by the genetics of the cow and embryo, and by health, nutritional, endocrine, environmental factors affecting ovarian and uterine biology of the cow. Although embryonic losses in the first week are substantial, failures in the peri-implantation stages of conceptus development seem to account for an important portion of pregnancy losses (Fig. 1A). It has been estimated that 39% of day 6 morulas fail to survive by day 28 of development (Ribeiro, 2018). The moderate efficiency of embryo transfer (ET) as breeding strategy for lactating cows further emphasize the significance of the peri-implantation period for pregnancy success (Table 1).

In cattle, the developing morula enters the uterus around day 4 but implantation starts only around day 20. Therefore, formation of the blastocyst, expansion and hatching from the zona pellucida, formation of ovoid conceptus, elongation and initial differentiation of trophectoderm binucleated cells must all be coordinated by uterine histotroph. Over the years, substantial research efforts have been placed to understand the biology of these events and their connection to pregnancy failures in dairy cows. Large emphasis has been given to the elongation phase of conceptus development because of its complexity and necessity for maternal recognition of pregnancy and implantation. This review summarizes our current understanding of the physiological and cellular requirements for successful elongation of the conceptus and discusses the potential contribution of impaired elongation to subfertility of lactating dairy cows.

## Physiological and cellular requirements of elongation

Elongation of the preimplantation conceptus entails remarkable expansion of extraembryonic tissues along the uterine lumen in a short window of development (Betteridge, 1980; Wales and Cuneo, 1989). In cattle, elongation starts around day 14 and, within 3 days, the conceptus grows from <5 mm to approximately 250 mm in length and occupies almost the entire extension of the pregnant uterine horn. The exponential increase in tissue mass is explained mainly by rapid proliferation of trophectoderm cells (Wang *et al*., 2009). The augmented rate of proliferation is induced by driver signals and demands substantial supply of nutrients (e.g. lipids, amino acids, sugar, nucleotides) for energy expenditures and synthesis of biomass. The required signals and nutrients are provided by the uterine histotroph, whose composition is modulated by the activity of ovarian steroids and conceptus-derived molecules (Spencer *et al*., 2004). This section reviews scientific data that provide insights on the physiological and cellular events that coordinate conceptus elongation.

**Table 1:** Pregnancy per embryo transfer in lactating dairy cows according to the type of embryo^1^ .

Reference	Embryo	Frozen/Fresh	Recipient, n	Pregnant, n	Pregnant, %
Chebel *et al*., 2008	IVP	Fresh	524	269	51.30%
Stewart *et al*., 2011	IVP	Fresh	136	62	45.50%
Mikkola *et al*., 2015	IVP	Fresh	2935	1429	48.70%
Monteiro *et al*., 2015	IVP	Fresh	360	96	26.60%
Ferraz *et al*., 2016	IVP	Fresh	2225	723	32.50%
Pereira *et al*., 2016	IVP	Fresh	2003	837	41.80%
Pereira at al., 2017	IVP	Fresh	323	100	31.00%
Barbosa *et al*., 2018	IVP	Fresh	1097	396	36.10%
Reese *et al*., 2018	IVP	Fresh	240	80	33.30%
***Total Fresh IVP***			***9843***	***3992***	***40.60%***
Chebel *et al*., 2008	IVP	Frozen	109	48	44.00%
Block *et al*., 2010	IVP	Frozen	142	47	33.10%
Stewart *et al*., 2011	IVP	Frozen	178	56	31.60%
Marinho *et al*., 2015	IVP	Frozen	3392	1241	36.60%
Ferraz et al., 2016	IVP	Frozen	194	56	28.90%
***Total Frozen IVP***			***4015***	***1449***	***36.10%***
Pereira *et al*., 2013	SOV	Fresh	487	216	44.40%
Ferraz *et al*., 2016	SOV	Fresh	651	303	46.50%
***Total Fresh SOV***			***1138***	***519***	***45.60%***
Thatcher *et al*., 2001	SOV	Frozen	181	76	42.00%
Kenyon *et al*., 2012	SOV	Frozen	289	84	29.10%
Kenyon *et al*., 2013	SOV	Frozen	135	49	36.20%
Mikkola *et al*., 2015	SOV	Frozen	7762	3283	42.30%
Ferraz *et al*., 2016	SOV	Frozen	1042	356	34.20%
***Total Frozen SOV***			***9409***	***3849***	***40.90%***
**Total**			**24405**	**9808**	**40.20%**

1 IVP = *in vitro*-produced embryo. SOV = *in vivo*-produced embryo.

## Priming of the uterus by ovarian steroids

During the course of the estrous cycle, endometrium physiology in ruminants is regulated by changes in concentrations of ovarian steroids in blood circulation and temporal changes in expression of steroid receptors in endometrial cells (Spencer and Bazer, 1995). Activity of steroid hormones in endometrial cells determines the length of the estrous cycle and is essential for pregnancy establishment and maintenance (Thatcher *et al*., 1989, Geisert *et al*., 1992). Classic studies that use replacement of ovarian steroids in ovariectomized ewes were fundamental to demonstrate the importance of these hormones to early conceptus development. Ewes ovariectomized 3.5 days after breeding (Foote *et al*., 1957) or at the time of embryo transfer 4 days after estrus (Moore and Rowson, 1959) were able to support pregnancy to term when exogenous progesterone was administered from the time of the ovariectomy until day 60 of gestation. To be successful, daily doses of at least 10 mg of progesterone were required (Moore and Rowson, 1959; Bindon, 1971). It was observed that exogenous progesterone increased cell height of glandular and luminal epithelial cells of the endometrium (Bindon, 1971) and increased their secretory activity into the uterine lumen (Miller and Moore, 1976; Garrett *et al*., 1988).

**Figure 2:**
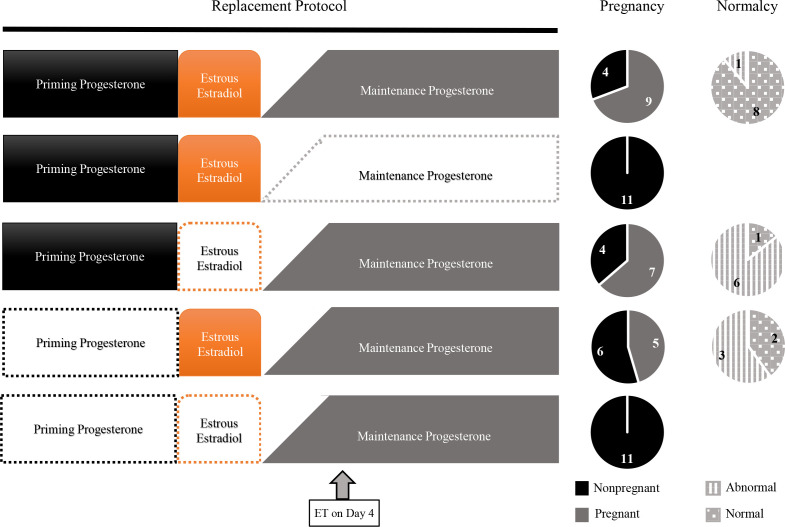
Survival and quality of ovine conceptuses transferred to ovariectomized ewes subject to steroid replacement protocols. Forms without color within the protocol represent the omission the specific treatment from the protocol. Data from Miller and Moore (1976).

Miller and Moore (1976) established a steroid replacement protocol for ovariectomized ewes that would resemble endogenous ovarian secretion during early pregnancy (referred to as maintenance progesterone), around the time of estrus (referred to as estrous estradiol) and during the luteal phase immediately preceding estrus (referred to as priming progesterone). This protocol was successful to support embryo development after ET on day 4. Overall, 72% of the recipient ewes (73 out of 101) had a conceptus on day 25, and 62% of the conceptuses (45 out of 73) were considered morphologically normal. The importance of each component of the established protocol was also investigated (Fig. 2). The complete protocol resulted in 9 pregnant and 8 normal conceptuses out of 13 ewes. As expected, pregnancies were not obtained (0 out of 11) when the maintenance progesterone was omitted. Omission of the estrous estradiol resulted in smaller number of pregnancies (7 out of 11) and increased proportion of abnormal conceptuses (6 out of 7). Similarly, exclusion of the priming progesterone reduced the number of pregnancies (5 out of 11) and increased the proportion of abnormal conceptuses (3 out of 5). Finally, the omission of both priming progesterone and estrous estradiol resulted in no pregnancies (0 out of 11). Investigation of endometrium metabolism revealed that omission of maintenance progesterone reduced synthesis of proteins and RNA to DNA ratio, and omission of estrous estradiol and priming progesterone had additive effects reducing the RNA to DNA ratio. Thus, not only progesterone during the time of conceptus development but also estradiol secretion at time of estrus and the progesterone secreted before estrus have important roles in the establishment of a uterine environment suitable for normal development of embryos. These results and interpretation were reinforced by a second experiment (Miller *et al*., 1977).

The development of high-throughput technologies for transcriptomics has allowed researchers to revisit classic concepts in reproductive biology and expand our understanding of the molecular control of reproduction. For instance, Shimizu *et al*. (2010) used ovariectomized cows to investigate the independent and the interdependent effects of progesterone and estradiol in the endometrium transcriptome. Compared to a control group (no additional treatment), replacement of progesterone alone, estradiol alone, and the combo treatment resulted in 289, 721, and 689 differentially expressed genes (DEG) in the endometrium, respectively. Interestingly, there was little overlap in the endometrial response to progesterone alone compared with the response observed in groups that received estradiol. Contrarily, the response to estradiol alone and the response to progesterone and estradiol combined had 50% overlap. Functional analyses of DEG revealed that the response of the endometrium to combined administration of the two hormones did not simply reflect the net effect of individual treatments, but rather suggested a functional interaction between the two hormones. In general, priming of the endometrium with progesterone caused the amplification of some estrogen- specific responses and created novel transcript responses to the estradiol treatment. Among the progesterone- primed estrogen response were genes involved with cell differentiation, migration and adhesion.

Transcriptome studies of the bovine endometrium were also fundamental to understand the direct regulation of endometrial physiology by progesterone during the time of conceptus development. The magnitude of progesterone effects on endometrium physiology was evidenced by the substantial temporal changes in the transcriptome of endometrial cells at different stages of the estrous cycle (Bauersachs *et al*., 2005; Mitko *et al*., 2008; Forde *et al*., 2009, 2011) and its modulation by exogenous progesterone supplementation (Forde *et al*., 2009, 2011). These studies reported multiple patterns of gene expression throughout the estrous cycle, with both up- and down- regulation of specific transcripts caused by exposure of the endometrium to increasing concentration of circulating progesterone. These changes are likely important for the preparation of an optimal uterine environment for the onset of conceptus elongation, and were frequently associated with transport of molecules, carbohydrate and lipid metabolic processes, extracellular matrix remodeling, growth factors and cytokine signaling, and immune responses. To some extent, these changes observed naturally during the estrous cycle are advanced by the supplementation of exogenous progesterone between days 3 and 8 of the estrous cycle (Forde *et al*., 2009, 2010, 2011; Okumu *et al*., 2010). It is noteworthy that changes in endometrial physiology are generally amplified around day 13 of the estrous cycle with the downregulation of progesterone receptors in epithelial cells, which has important implications for histotroph secretion (Spencer and Bazer, 1995; Okumu *et al*., 2010).

The temporal regulation of endometrium physiology affects the composition of the uterine histotroph which is important to meet the evolving cellular requirements of the developing conceptus. Embryo transfer studies have clearly demonstrated the importance of synchrony between donor and recipient for successful establishment of pregnancy (Moore and Shelton, 1964), which is intrinsically associated with the ability of the uterus to provide an adequate uterine environment to a specific stage of development. As the estrous cycle progresses, changes in the uterine environment support the formation of an ovoid conceptus by day 13 and provide signals that augment proliferation of trophectoderm cells and the onset of elongation. For instance, Mullen *et al*. (2012a) compared the proteomics of uterine flushings collected from dairy heifers on days 7 and 13 of the estrous cycle and identified 38 proteins with different abundances. These proteins were associated with tissue remodeling, metabolism, and free radical management, and might be relevant for the onset of elongation.

The composition of histotroph is also affected by temporal changes in the ability of the endometrium to sequestrate and transport metabolites into the uterine lumen. For instance, concentrations of amino acids in the uterine fluid changes from early to mid-diestrus (Hugentobler *et al*., 2007) and are altered by the profile of steroid hormones during the peri-ovulatory period (França *et al*., 2017). Transcript expression of the facilitative glucose *SLC5A1* in endometrial epithelium of heifers was greater on day 13 of the estrous cycle compared with samples collected on days 5 and 7 (Forde *et al*., 2010). This transporter could facilitate the influx of glucose into the uterine lumen according to the demand of the developing conceptus. Similarly, transcript expression in the endometrium and protein concentration in the histotroph of retinol-binding protein 4 (RBP4) were increased on day 13 compared with day 7 of the estrous cycle in heifers (Mullen *et al*., 2012b), which might be important to regulate sequestration of circulating retinol and its availability in the uterine lumen at the onset of elongation. In fact, Costello *et al*. (2010) reported greater concentration of retinol in uterine flushings collected at late-diestrus compared with those collected in metaestrus and early diestrus. Moreover, transcript expression of insulin-like growth factor (IGF) binding protein 1 (*IGFBP1*) in the endometrial epithelium of heifers and cows was undetectable on days 8-10 but highly expressed on days 12-14 of the estrous cycle, which could influence sequestration and bioavailability of IGF1 in the uterine lumen, in addition to its IGF-independent roles in conceptus elongation (Robinson *et al*., 2000; Simmons *et al*., 2009).

Steroid hormones also influence lipid metabolism in endometrial cells. The amount of lipid droplets in the epithelium fluctuates according to the phase of estrous cycle in both, cows (Wordinger *et al*., 1977) and ewes (Brinsfield and Hawk, 1973), being low during metaestrus and increasing during diestrus. Brinsfield and Hawk (1973) administered 25 mg of exogenous progesterone for 5 days in ovariectomized ewes and observed an accumulation of lipids in the endometrium that was comparable to the accumulation observed in a spontaneous estrous cycle, thus concluding that progesterone was the main factor inducing the accumulation of lipids during diestrus. The mechanism by which progesterone influences the formation of lipid droplets is still unknown, but genes reported to be modulated by progesterone in the endometrium of cattle such diacylglycerol O- acyltransferase 2 (*DGAT2*) and lipoprotein lipase (*LPL*) might be important in this process. Transcript expressions of *DGAT2* and *LPL* in epithelial cells are increased and decreased, respectively, during diestrus (Forde *et al*., 2009, 2010, 2011). Their encoded proteins have opposing functions, with the former being involved in synthesis of triglycerides and the later in hydrolysis of triglycerides. Neutral lipids such as triglycerides facilitate the packing of lipids and the formation of lipid droplets (Thiam *et al*., 2013).

## Reprogramming of conceptus cells at the onset of elongation

The specific components of the histotroph that drive conceptus elongation are not completely known. The nature of these components could be potentially identified by evaluating the biology of conceptus cells at the onset of elongation and how they sense and respond to the histotroph stimuli. Recently, two independent studies evaluated the transcriptome of bovine conceptuses at early stages of elongation and revealed substantial changes in cell biology with the onset of elongation (Barnwell *et al*., 2016; Ribeiro *et al*., 2016a). Comparison of DEG in the two studies shows ample overlap, which increases the confidence on the methodology used and in the results obtained. Functional analyses of DEG not only revealed important pathways that are modulated during the onset of elongation but also identified potential upstream regulators of the observed changes in the transcriptome, which might represent the uterine signals required for elongation. Changes in the transcriptome of conceptus cells with the magnitude observed at early stages of elongation do not seem to occur at later stages of elongation (Valour *et al*., 2014). Thus, the substantial change in transcriptome at the onset of elongation is suggested here as a reprogramming event of extraembryonic cells required for successful elongation. To some extent, the main findings of these two recent studies are also supported by previous research evaluating the development of bovine conceptuses but using distinct experimental designs and methodology (Hue *et al*., 2007, 2012; Clemente *et al*., 2011; Mamo *et al*., 2011).

A considerable portion of the DEG reported by Barnwell *et al*. (2016) and Ribeiro *et al*. (2016a) referred to cytoskeleton organization and cell adhesion molecules, and reinforced the idea that elongation in ruminants is not the simple proliferation of cells that are molded according to the shape of the uterine lumen but an elaborate event that requires cytoskeleton reorganization and active interaction between cells and the extracellular matrix (Kim *et al*., 2010). Ribeiro *et al*. (2016a) suggested that with the onset of elongation, the upregulation of multiple transmembrane cell-matrix adhesion proteins increases the capacity of conceptus cells to interact with the extracellular matrix and endometrial epithelial cells, receiving mechanical load and transmitting it to intracellular actin filaments. Enhanced expression of myosins and actin crosslinker proteins would aid actin filaments to generate intracellular mechanical force that could be used for cell motility and force-generated migration. Candidate genes to coordinate these events were identified and include transmembrane 4 L six family member 1 (*TM4SF1*), transgelin (*TAGLN*), and ankyrin repeat domain 1 cardiac muscle (*ANKRD1*).

Ribeiro *et al*. (2016a) also reported IGF1 and protein kinase AKT as upstream regulators of transcriptome changes in conceptus cells during the onset of elongation, both with predicted increased activity. Insulin-like growth factor 1 is a mitogen molecule whose main downstream effects are stimulation of cell proliferation and cell survival. The AKT kinase is not only an essential component in the downstream signaling of the IGF1 receptor (Le Roith, 2001) but is also part of the mammalian target of rapamycin (mTOR) signaling pathway, which has been described to be important for cytoskeletal changes, adhesion and migration of ovine trophectoderm cells (Kim *et al*., 2010). Synthesis and secretion of IGF1 is stimulated by growth hormone (GH) in multiple tissues, although the liver is responsible for production of most of the circulating IGF1 (Le Roith, 2001). The bovine endometrium expresses GH receptor, IGF1, and IGF1 receptor (Robinson *et al*., 2000; Rhoads *et al*., 2008), while the preimplantation bovine conceptus expresses both GH and IGF1 receptors (Yaseen *et al*., 2001; Sawai *et al*., 2007). Therefore, endometrial physiology and the developing conceptus can be affected by systemic GH and IGF1 as well as by the locally produced endometrial IGF1. Of note, the increased expression of *IGFPB7* in conceptus cells during elongation is one of the most consistent outcomes reported in multiple studies (Hue *et al*., 2012; Barnwell *et al*., 2016; Ribeiro *et al*., 2016a). Moreover, the elongating bovine conceptus also seems to express IGFBP1 and IGFBP3 (Sawai *et al*., 2007; Ribeiro *et al*., 2016a).

**Figure 3:**
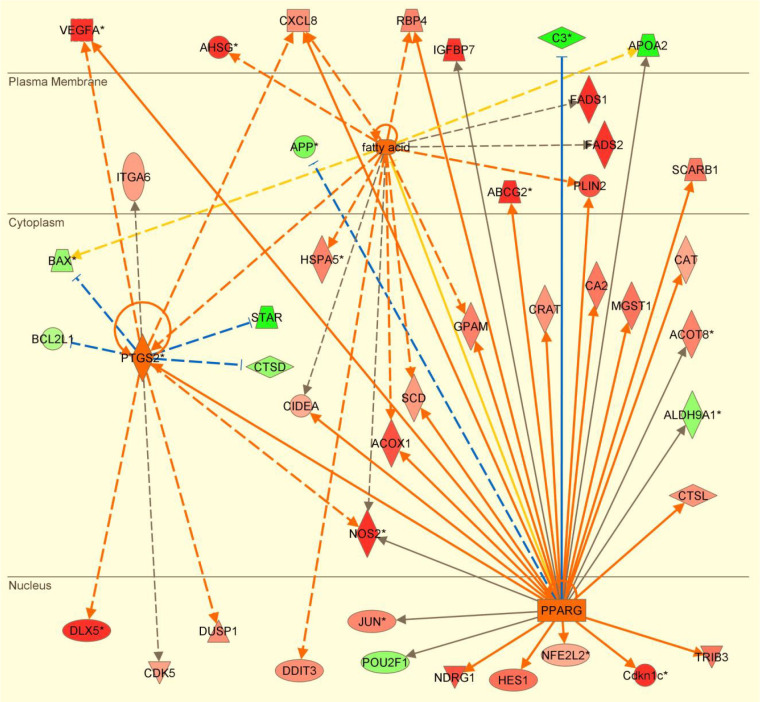
Upstream regulators (orange) and changes in transcriptome (red = upregulated gene; green = downregulated gene) associated with lipid metabolism during the onset of elongation of the bovine conceptus. Data from Ribeiro *et al*. (2016a).

Lipid metabolism was one of the top molecular and cellular functions associated with the DEG in the two studies described above (Barnwell *et al*., 2016; Ribeiro *et al*., 2016a). In fact, Ribeiro *et al*. (2016a) identified at least 132 genes with known annotation and linkage to this specific function. Among those genes, some were involved with lipid uptake, lipid droplet formation, biogenesis of peroxisomes, activation, oxidation, desaturation and elongation of fatty acids, biosynthesis of phospholipids, mobilization of membrane phospholipids, biosynthesis of prostaglandins, and transport of prostaglandins and other lipids metabolites (Fig. 3). In addition, peroxisome proliferator activated receptor gamma (*PPARG*) not only had transcript expression markedly increased during the onset of elongation but was also listed as an important upstream regulator of the transcriptome changes observed in trophectoderm cells during elongation. In fact, PPARG is a nuclear receptor that functions as ligand-dependent transcription factor and its transcript expression was highly correlated with the expression of other genes known to be involved with lipid metabolism and conceptus development in ruminants. The increase in expression of PPARG in conceptus cells during elongation is likely caused by the accumulation of lipids in the uterine lumen during diestrus (Ribeiro *et al*., 2016d), although a direct effect of progesterone on conceptus cells cannot be discarded (Kim *et al*., 2008; Yang *et al*. 2016).

The PPARG have large binding pockets that interact promiscuously with multiple lipid ligands including unsaturated fatty acids and prostanoid metabolites (Kliewer *et al*., 1997; Nagy *et al*., 1998). Itoh *et al*. (2008) examined crystal structures of PPARG bound to oxidized unsaturated fatty acids and concluded that the large binding pocket of the receptor confers remarkable versatility in ligand binding and could therefore act a cellular sensor of the varying composition of the cellular pool of fatty acid ligands. Binding of fatty acids into the ligand pocket of PPARG causes conformational changes in the receptor that facilitates the formation of heterodimers with retinoid X receptor (RXR) and subsequent binding of the dimer to PPAR response elements (PPRE) in regulatory regions of target genes (Berger and Moller, 2002). Putative PPRE were identified in regulatory regions of several genes transcriptionally regulated during the onset of conceptus elongation, which suggests a direct effect of PPARG on the abundance of the respective transcripts (Ribeiro *et al*., 2016a). Among genes with PPRE in regulatory regions is PPARG, which suggests that activation of PPARG could alter its own expression (Ribeiro *et al*., 2016a, d).

The activity of the nuclear receptor dimer PPARG-RXR as transcription factor depends on the presence and binding of their respective ligands. The increased expression of RBP4 and accumulation of lipid droplets in the bovine endometrium during diestrus likely contribute for this requirement (Wordinger *et al*. 1977; Mullen *et al*., 2012b). In fact, retinol and lipids are constitutive part of the histotroph of cows on day 15 of the estrous cycle (Costello *et al*., 2010; Ribeiro *et al*., 2016a). Moreover, transcript expression of *RBP4* and fatty acids transporters such as *SCL27A6* increases in conceptus cells during elongation, which might facilitate capture of retinol and fatty acids from histotroph (Ribeiro *et al*., 2016a). Research in sheep has shown that the elongating conceptus accumulates lipid droplets within the cytoplasm of trophectoderm cells (Carnegie *et al*., 1985) and causes a reduction in lipid droplet accumulation on endometrial epithelial cells (Boshier *et al*., 1987), suggesting the existence of an active transfer of lipids from the endometrium to the conceptus. Lipids would be used not only for control of gene expression but also for synthesis of biomass, energy, and cell signaling (Ribeiro *et al*., 2016d).

The idea that PPARG is important for elongation of the bovine conceptus is strongly supported by an elegant study performed in sheep. Brooks *et al*. (2015) performed a loss-of-function study by infusing morpholino antisense oligonucleotides in the uterus of pregnant ewes from day 7 to day 14 after breeding using an osmotic pump affixed surgically in the mesosalpinx. Morpholino antisense oligonucleotides for PPARG resulted in the recovery of growth-retarded conceptuses, while infusion of the designed control or PPAR-delta morpholinos resulted in normally elongated conceptus on day 14. Binding sites of PPARG in ovine conceptus cells were identified and several of them were in close proximity to genes involved in lipid biosynthesis and metabolism also reported to be differently regulated during onset of elongation of bovine conceptus (Ribeiro *et al*., 2016a). Altogether, these studies highlight the importance of lipid metabolism for the onset of conceptus elongation.

## Intensification of the crosstalk between conceptus and endometrium

Physiology of the bovine endometrium is not only influenced by ovarian steroids but also by paracrine effects of conceptus-derived bioactive products. The communication between developing conceptus and endometrium seems to start as early as day 7 of development (Sponchiado *et al*., 2017) and is intensified with the elongation of the conceptus and consequent accumulation of conceptus-derived products in the uterine lumen (Forde *et al*., 2011; Bauersachs and Wolf, 2012). The highly active trophectoderm of the elongating conceptus secrete products such as interferon-τ (IFNT) and prostaglandins that modulate endometrial physiology, establishing a complex crosstalk between the two tissues that coordinate the continuous growth of the conceptus, maternal recognition of pregnancy and maintenance of the corpus luteum, increase blood flow to the pregnant uterus, establishment of uterine receptivity to implantation, and formation of a functional placenta (Spencer *et al*., 2004, 2013; Bauersachs *et al*., 2012; Pinaffi *et al*., 2017). The complete molecular crosstalk between the two tissues is, however, more complex and not fully understood. Mamo *et al*. (2012) evaluated transcriptome data from bovine conceptuses and endometria and identified a total of 133 conceptus ligands that could interact with corresponding receptors on the endometrium, and 121 endometrium ligands that could interact with corresponding receptors on the conceptus.

The composition of the histotroph during elongation also changes dramatically as a result of conceptus secretions and dynamic responses from the endometrium, and these changes are essential to meet the requirements of the expanding conceptus (Spencer *et al*., 2004). The changes in composition reported in cattle include amino acids (Groebner *et al*., 2011), proteins (Forde *et al*., 2014) and lipids (Ulbrich *et al*., 2009; Ribeiro *et al*., 2016a), and are supported by similar reports in sheep (Gao *et al*., 2009; Romero *et al*., 2017). For instance, Groebner *et al*. (2011) reported a pregnancy-dependent increase in the concentration of all essential amino acids, except for methionine, during the elongation phase. Similar results were observed for the concentrations of prostaglandins (Ulbrich *et al*., 2009). Moreover, Ribeiro *et al*. (2016a) reported reduced amounts of arachidonate, but increased amounts of arachidonate-derivative molecules in uterine flushing of pregnant cows compared with those of nonpregnant cows on day 15. The arachidonate-derivative molecules have been described as ligands of PPARG and may be important for coordination of gene expression in conceptus cells during elongation.

A recent study described a negative association between pregnancy success in dairy cows and size of the uterus (Baez *et al*., 2016). Cows with bigger uterus had reduced P/AI compared with cows with smaller uterus. Although the reasons for such difference in fertility are unknown, the large uterine volume and the spatial regulation of endometrium physiology (Sponchiado *et al*., 2017) might complicate the crosstalk between the developing conceptus and the endometrium and be ineffective to block luteolysis. In addition, two companion studies (Geary *et al*., 2016; Moraes *et al*., 2018) suggested that impaired crosstalk between the elongating conceptus and endometrium contribute to subfertility of beef heifers. In their experimental model, heifers classified as high-fertile and those classified as subfertile presented similar development of ovoid conceptuses by day 14, but remarkable differences in size and transcriptome of conceptuses and in transcriptome of pregnant and nonpregnant endometria on day 17. The reported transcriptome differences in both tissues did not seem to be related exclusively to the observed differences in size of the conceptus but also associated with the capacity of the endometrium to respond to conceptus-derived factors.

## Elongation of conceptus in lactating dairy cows

Years of genetic selection for milk production have intensified the homeorhetic control of dairy cow’s metabolism to support lactation (Bauman and Currie, 1980). Similar to high-performance athletes, modern high-producing dairy cows have remarkable nutrient demands, with total requirements averaging 4 times the maintenance requirements (Santos *et al*., 2010). Around parturition, however, feed intake is usually depressed and the caloric and nutrient requirements of the cow postpartum are only partially met by feed consumption, causing extensive mobilization of nutrients from body tissues (Drackley, 1999). Adipose tissue is particularly affected by reduced concentrations of glucose and insulin that up-regulate lipolytic signals for hydrolysis of stored triglycerides and increase availability of nonesterified fatty acids (NEFA) to be used as an energy source. The imbalance in energy is extended to nutrients such as amino acids, minerals and vitamins (Goff, 2004; LeBlanc *et al*., 2004). Reduced insulin also impacts the expression of GH receptors in the liver and consequently the circulating concentrations of IGF1 (Butler *et al*., 2003). In addition, the elevated feed intake required for high milk yields result in augmented blood flow to the liver, increased metabolism and reduced concentrations of circulating steroid hormones (Wiltbank *et al*., 2006). All these features have implications for reproductive biology and are often associated with the subfertility presented by lactating dairy cows (Chagas *et al*., 2007). Studies investigating potential risk factors for failures in conceptus development and elongation in lactating dairy cows are discussed below.

## Lactation

Multiple studies have investigated the potential impact of lactation on the development of the preimplantation conceptus (Table 2). Their experimental designs included the comparison of conceptus development and endometrial physiology between cows and heifers (Valour *et al*., 2014; Bauersachs *et al*., 2017; Forde *et al*., 2017) and between lactating and non- lactating cows (Cerri *et al*., 2012; Maillo *et al*., 2012; Thompson *et al*., 2012; Bauersachs *et al*., 2017; Forde *et al*., 2017). The latter approach would randomly assign cows to be dried-off right after calving or to continue to be milked until completion of the experiment. The overall hypothesis in these studies was that lactation would cause substantial changes in the metabolism of cows that would alter the uterine environment and impaired conceptus development.

Studies comparing lactating (L) and nonlactating (NL) cows were successful to create differences in metabolism, which included lower concentrations of glucose, insulin, and IGF1, and higher concentrations of NEFA and ketones in serum of L compared with NL during the postpartum period (Maillo *et al*., 2012; Thompson *et al*., 2012). These studies, however, failed to show differences in conceptus development (Table 2). Thompson *et al*. (2012) examined conceptuses on day 17 after timed AI and observed similar length and no differences in gene expression of selected genes. Maillo *et al*. (2012) examined conceptuses on day 14 after transfer of multiple in vitro-produced embryos on day 7 and did not observe differences in conceptus survival or size. Forde *et al*. (2017) investigated the transcriptome of conceptuses and the amino acid composition of uterine fluid in L and NL on day 19 of pregnancy but no differences were found. Two studies compared the transcriptome of intercaruncular endometrial cells of L and NL cows (Cerri *et al*., 2012; Bauersachs *et al*., 2017). Cerri *et al*. (2012) used endometrium from pregnant and nonpregnant cows on day 17. Using a less stringent cutoff (nominal P value ≤ 0.01), they reported 277 DEG mainly involved with immune responses, cell adhesion and tissue remodeling. Bauersachs *et al*. (2017) compared endometrium of L and NL cows on day 19 of pregnancy after ET on day 7. Using adjusted P value ≤ 0.10 as cutoff, only 28 transcripts were considered differently expressed, and none of them were reported as DEG by Cerri *et al*. (2012). It is important to mention that differences in progesterone concentration in serum between L and NL cows were observed only in the study by Cerri *et al*. (2012) and not in the study by Bauersachs *et al*. (2017), which might also contribute for the slight different outcomes.

Studies comparing transcriptome of conceptus and endometrium between L cows and nulliparous heifers (NH) reported considerable differences (Table 2). The DEG in endometrium reported by Bauersachs *et al*. (2017) were mostly involved with immune responses and cell adhesion and migration. Based on gene expression, Forde *et al*. (2017) predicted that conceptus from NH were more advanced compared with those recovered from L cows. Conversely, Valour *et al*. (2014) found no differences in development based on length of the conceptuses and morphological and molecular staging of the embryonic discs. Nonetheless, 483 transcripts were differently expressed in trophectoderm cells and revealed important differences in lipid and energy metabolism. Although the comparison between L and NH has many confounding factors, the results of these investigations are important to characterize developmental biology in cattle and how it might be affected by distinct uterine environments.

**Table 2:** Impact of lactation and parity on survival, size, and transcriptome of conceptuses and on transcriptome of endometria in dairy cattle.

Item	Lactating cow	Nonlactating cow	Heifer	Reference
Survival of conceptus on day 14, % (n/n)^1^	39.8 (67/175)	33.3 (65/175)	---	Maillo *et al*., 2012
Length of conceptuses on day 14 , mm	1.6 ± 0.5	1.2 ± 0.5	---	Maillo *et al*., 2012
Pregnant on day 17 after AI, (n/n)	80 (8/10)	50 (6/12)	---	Thompson *et al*., 2012
Length of day 17 conceptuses, mm	251 ± 51	200 ± 72	---	Thompson *et al*., 2012
DEG in day 17 conceptuses ^2^	Reference	0	---	Thompson *et al*., 2012
DEG in day 19 conceptuses	Reference	0	269	Forde *et al*., 2017
DEG in day 19 pregnant endometria	Reference	28	238	Bauersachs *et al*., 2017
DEG in day 17 pregnant endometria	Reference	277	---	Cerri *et al*., 2012
DEG in day 18 conceptus	Reference	---	483	Valour *et al*., 2014
Fully elongated conceptus^3^ , % (n/n)	72.7 (8/11)	---	63.6 (7/11)	Valour *et al*., 2014
Length of day 18 conceptuses (elongated), mm	157 ± 14	---	151 ± 11	Valour *et al*., 2014
Length of day 18 conceptuses (delayed), mm	67 ± 9	---	58 ± 22	Valour *et al*., 2014

1 Recipients received multiple *in vitro*-derived embryos on day 7.

2 Analyses of candidate genes performed by PCR only.

3 Larger than 150 mm in length.

## Diseases postpartum

Clinical diseases caused by microbial infection and tissue injury are prevalent in postpartum dairy cows and have consequences for reproduction (Santos *et al*., 2010; Ribeiro *et al*., 2013, 2016b). Approximately 40% of dairy cows have at least one clinical disease between calving and the first breeding postpartum, and the odds of pregnancy per AI (P/AI) after synchronized ovulation are reduced by 30% in cows affected by clinical diseases postpartum compared with those with no clinical disease (Ribeiro and Carvalho, 2017). Moreover, conception risk is also reduced by clinical diseases in cows receiving a viable embryo on day 7 of the estrous cycle, which indicates that altered uterine environment must account for at least part of the subfertility of cows diagnosed with clinical diseases postpartum (Table 3; Ribeiro *et al*., 2016b).

To evaluate the impact of diseases on conceptus elongation, health information of 148 lactating cows was collected from parturition until first postpartum AI, and uterine flushing for recovery of conceptuses was performed 15 or 16 days after AI. Cows with disease had shorter conceptuses and reduced concentration of IFNT in the uterine flush (Ribeiro *et al*., 2016b). These results were supported by a second experiment that evaluated the transcript expression of IFN stimulated genes (ISGs) in peripheral blood leukocytes (PBL) on day 19 after AI (Ribeiro *et al*., 2016b). As expected, the expression of ISGs was about 2-fold greater in cows later diagnosed pregnant. However, an interaction between pregnancy status and disease category was detected. A significant increase in expression of ISGs by pregnancy was only observed in cows that did not have disease and not in those that had disease before AI. Although expression of ISGs does not depend exclusively of stimulation by IFNT, it has been used by researchers as an indirect method to evaluate conceptus development without the need to terminate pregnancy (Ribeiro *et al*., 2014b, 2016b).

Comparison of the transcriptome of conceptuses recovered from cows having or not non-uterine diseases before AI revealed small but interesting differences in transcript expression (Ribeiro *et al*., 2016b). Conceptus recovered from cows that had disease before AI presented upregulation of genes associated with inflammatory response, and inflammatory molecules such as lipopolysaccharide, tumor necrosis factor ɑ (TNFɑ), and IFN-γ were predicted as potential upstream regulators of such differences. In addition, the predicted downstream effects of the transcriptome changes were activation of immune cells and tissue rejection, which in the context of the pregnant uterus could be translated to activation of the maternal immune system in the endometrium and rejection of the allogeneic conceptus.

Altogether, these findings reinforce that disease postpartum is a relevant problem in dairy herds and has a substantial impact on reproduction. It also suggest that elongation of the preimplantation conceptus is impaired in this subgroup of cows and might help explain the difference observed in P/AI and the higher incidence of late embryonic and fetal losses. It is still not clear how diseases occurring early in the postpartum would have a long term impact on uterine environment, but it has been suggested that clinical diseases would lead to exacerbated inflammation and long-lasting effects on energy and lipid metabolism that could influence the composition of the uterine histotroph and consequently conceptus development (Ribeiro, 2018).

**Table 3:** Reproductive outcomes of first breeding postpartum in dairy cows according to incidence of disease before breeding and breeding technique^1^ .

Item	Pregnant day 45 (%)	Calving (%)	Pregnancy loses (%)
-------- Adjusted mean ± SEM^2^ --------			
No disease-AI	38.8 ± 1.8	32.9 ± 1.7	12.4 ± 1.5
Disease-AI	31.0 ± 2.1	22.2 ± 1.9	21.3 ± 3.1
No disease-ET	40.7 ± 1.7	35.9 ± 1.7	11.1 ± 1.5
Disease-ET	35.9 ± 2.4	28.2 ± 2.2	22.4 ± 3.4
P-value			
Disease	<0.01	<0.01	<0.01
Breeding technique	0.12	0.03	0.27
Disease x breeding technique	0.37	0.27	0.59
Adjusted odds ratio (confidence interval)^3^			
Within AI	0.71 (0.58-0.87)	0.58 (0.46-0.73)	1.92 (1.24-2.98)
Within ET	0.82 (0.65-1.02)	0.70 (0.55-0.90)	2.30 (1.41-3.76)

1 Data from Ribeiro *et al*. (2016b).

2 Adjusted mean and standard error of the mean for cows that had or not disease before breeding and were bred by artificial insemination (AI) or embryo transfer (ET).

3 Adjusted odds ratio (confidence interval) for the effect of disease within cows bred by AI and within cows bred by ET.

## Anovulation at the onset of synchronization programs

Anovulation is a normal and temporary physiological condition of dairy cows during early postpartum. It is characterized by lack of regular estrous cycles and ovulation, although follicle growth is still present (Wiltbank *et al*., 2002). Time for resumption of estrous cyclicity postpartum varies among cows and is directly associated with energy balance in the first weeks of lactation (Butler, 2003). As consequence, 18 to 43% of dairy cows remain anovular at the end of the voluntary waiting period, constituting an important problem in achieving adequate reproductive performance in dairy herds (Rhodes *et al*., 2003; Santos *et al*., 2009). Adoption of timed AI programs maximizes submission to AI and lessens the problem of anovular cows reducing reproductive performance. Nevertheless, P/AI of anovular cows after synchronized estrus or ovulation is reduced compared with that of estrous cyclic herdmates independent of the breed of the cow (Santos *et al*., 2009; Ribeiro *et al*., 2016c).

Submission of anovular cows to synchronization programs creates a novel endocrine scenario where the ovulatory follicle develops under low concentration of progesterone (Bisinotto *et al*., 2010). When ovulation is successfully induced in anovular dairy cows by administration of GnRH, the newly formed corpus luteum (CL) secretes progesterone but it takes approximately 4 days for circulating concentrations to be above 1 mg/ml. As the interval between GnRH and PG shots in most synchronization programs is 7 days, priming progesterone in these cows would act for only 3 days. In the ovariectomized sheep model, priming progesterone replacement need to last at least 6 days in order to be effective (Moore, 1985). Thus, synchronization of anovular cows might represent an alternative model for insufficient priming progesterone with consequences to fertility. In fact, supplementation of progesterone in cows without a CL at the onset of synchronization program improves P/AI (Bisinotto *et al*., 2013). Moreover, priming progesterone is also important for estrogen-induced estrous behavior (Schinckel, 1954; Allrich, 1994) and, interestingly, anovular cows that express estrous behavior on the day of timed AI have P/AI comparable to that observed in estrous cyclic herdmates (Bisinotto *et al*., 2013; Ribeiro *et al*., 2014a). Thus, the requirements for priming progesterone may vary among individuals and estrous behavior might be seen as an indicator of sufficient exposure of the hypothalamus and endometrium to priming progesterone. Ribeiro *et al*. (2016c) compared conceptus elongation of anovular (HA) and estrous cyclic (HC) Holstein cows subjected to timed AI program. Considering the difference in P/AI between the two groups, it was initially predicted that HA cows would have poorly developed conceptuses. Surprisingly, conceptuses from HA cows were longer and secreted greater amounts of IFNT than conceptuses from HC cows. The difference in size of the conceptus was likely caused by the distinct progesterone profiles before and after AI. Reduced concentration of progesterone during the synchronization program allowed accelerated development of the dominant follicle, ovulation of a larger follicle and formation a larger CL after AI. The larger CL resulted in greater concentrations of progesterone in the postovulatory period, which is known to advance conceptus elongation (Lonergan and Forde, 2014). Nonetheless, HA cows had reduced concentrations of IGF1 in plasma, and their conceptuses presented remarkable differences in transcriptome. Although the difference in size of the conceptuses influenced the results (Ribeiro *et al*., 2016a), some of the altered transcripts seemed to be unrelated to elongation and suggest that conceptus cells from HA might be under greater cellular stress, which could be associated with greater pregnancy mortality after day 15 of development.

These findings taught important aspects of reproductive biology of anovular cows subjected to synchronization programs but are not conclusive regarding the causes of their subfertility. One could hypothesize that developmental problems in anovular cows occur before day 15 and the conceptuses that survived by day 15 would continue to develop comparably to conceptuses in estrous cyclic cows. In favor of this hypothesis are the phenotype of the conceptus and increased secretion of IFNT, and the potential effects of insufficient priming progesterone on oocyte quality (Santos *et al*., 2016) and early embryo development (Miller *et al*., 1977; Rivera *et al*., 2011). On the contrary, one could suggest that although conceptuses from anovular cows were longer and secreted more IFNT, they might not necessarily be better, and perhaps the difference in transcriptome could indicate imbalances in cell biology that could result in greater incidence of pregnancy loss after day 15. In support of the latter, research has demonstrated increased pregnancy loss in anovular cows (Santos *et al*., 2004) and in those that develop the ovulatory follicle under low concentrations of progesterone (Bisinotto *et al*., 2010). Unfortunately, we are unaware of data of anovular cows subjected to an ET program, which would isolate the potential effects on oocyte quality and early embryo development and inform whether the uterus contributes to the subfertility observed in this subgroup of cows.

## Concentrations of circulating ovarian steroids

The reduced concentration of circulating steroids in high-producing dairy cows caused by augmented metabolism and clearance of these hormones (Wiltbank *et al*., 2006) could compromise endometrium physiology and consequently conceptus development. In fact, there are extensive evidences in the literature that progesterone before estrus, estradiol during estrus and progesterone after estrus are associated with fertility in cattle and likely involve effects on endometrial physiology (Wiltbank *et al*., 2014; Madsen *et al*., 2015). For instance, recipient lactating cows that express estrus at the end of synchronization program have increased pregnancy per ET compared with those that did not present estrous behavior (32.7 *vs*. 46.2%; Pereira *et al*., 2016). As discussed above, estrous behavior is a result of adequate exposure of the hypothalamus to priming progesterone during the diestrus preceding estrus and adequate exposure to estrous estradiol. Thus, the reported differences in pregnancy success after ET demonstrate the importance of steroid hormones exposure prior breeding to endometrium physiology in the subsequent estrous cycle and conceptus development in lactating dairy cows. Moreover, faster rise in concentration of progesterone from day 0 to day 7 was also associated positively with pregnancy establishment following ET (Kenyon *et al*., 2013).

If concentration of steroid hormones is a critical factor for endometrial physiology and fertility of dairy cows, then supplementation of these hormones would be a reasonable strategy to overcome this problem. However, fertility responses of lactating cows to steroid supplementation are variable. Bisinotto *et al*. (2015) performed a meta-analysis to evaluate the impact of progesterone supplementation during timed AI programs, and reported an overall 8% increase in risk of pregnancy on day 32 and a tendency to reduce pregnancy losses after day 32. Conversely, supplementation of progesterone during metaestrus and early diestrus, although is the most effective and consistent method to promote conceptus elongation (Lonergan and Forde, 2014), has not proven to be consistent in increasing P/AI or P/ET in lactating dairy cows. In fact, recent large studies show limited success (Monteiro Jr *et al*., 2014) or even negative results (Parr *et al*., 2014; Monteiro Jr *et al*., 2015). In addition, supplementation of estradiol around estrus is generally successful to enhance estrous behavior but not P/AI in dairy cows (Sellars *et al*., 2006; Souza *et al*., 2007; Hillegass *et al*., 2008).

## Concentrations of circulating IGF1

**Figure 4:**
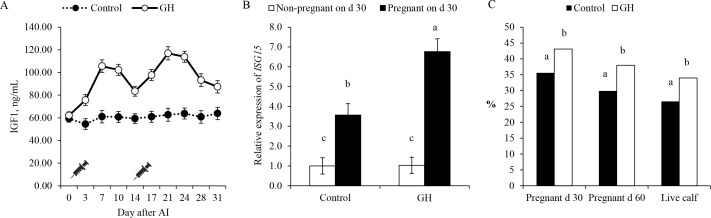
Impact of growth hormone (GH) supplementation on concentration of IGF1 in plasma (A), relative expression of ISG15 in leukocytes (B), and pregnancy and calving per AI (C) in lactating dairy cows. Data from Ribeiro *et al*. (2014b).

Ribeiro *et al*. (2014b) supplemented low doses of GH to lactating dairy cows during pre and peri- implantation periods, between days 0 and 28 relative to AI, with the objectives to increase circulating concentrations of IGF1 and improve conceptus development and survival. Supplementation of low doses of GH during the preimplantation period resulted in increased concentrations of IGF1 in plasma, improved P/AI, and reduced pregnancy losses, resulting in a 28% increase in calving per AI (Fig. 4). Conceptus development was evaluated by expression of ISG in PBL, concentration of pregnancy specific protein B (PSPB) in plasma, and ultrasonographic morphometry of conceptuses. Corroborating with the fertility results, supplementation of GH from days 0 to 28 after AI increased expression of ISGs in PBL, and resulted in earlier rise of PSPB in plasma of pregnant cows and larger conceptuses than untreated controls (Fig. 4). Altogether, these evidences support the role of IGF1 as an important mediator of preimplantation development and the potential use of pharmacological interventions to improve conceptus elongation and survival in lactating dairy cows.

## Genetics

A conceivably small but important portion of Jersey crossbred cows (CC) randomly selected in a grazing herd and subjected to the Ovsynch protocol. Compared to HC, CC cows presented advanced the variation in pregnancy success among dairy cows within and across herds is explained by the genetics of the cow, the genetics of the breeding sire, and the resulting genetics of the embryo (Santos *et al*., 2010; Butler, 2014; Cole *et al*., 2016; Han and Peñagaricano, 2016; Ortega *et al*., 2016). In general, the genetic heritability of reproductive traits is relatively low, generally less than 10%, and suggests that reproductive success is affect mainly by non-genetic factors. Nonetheless, the growing understanding of molecular regulation of reproduction will likely result in the development of new fertility traits and more efficient ways to selected high fertility cows. As depicted in Fig. 1B, conception risk in dairy cows has been low and stagnant for many years and therefore novel traits should target conceptus survival for consistent genetic improvements in conception risk. The contribution of genetics to the success of conceptus elongation, however, is still unknown. Some of the few studies exploring potential effect of genetics on elongation are discuss below.

Meier *et al*. (2009) compared the conceptus development and fatty acid composition of the endometrium in two strains of Holstein-Friesian cows, New Zealand (NZ) and North American (NA), managed in a pasture-based system. In general, NA cows have reduced P/AI compared with NZ cows and conceptus development could contribute for this difference in fertility. Reproductive tissues were obtained on day 17 of the estrous cycle or pregnancy. The average length of the conceptuses was similar between groups, but the variation in size was a 3.6-fold greater in those recovered from NA recipients (NZ: 20.8 ± 2.84 cm; NA: 27.9 ± 10.23 cm). Concentrations of C17:0 and C20:3n-3 were higher in the endometrial tissues from NZ cows, and there was a tendency for higher total concentrations of polyunsaturated fatty acids. Concentrations of C18:1, C20:2, and total monounsaturated fatty acids tended to be less in NZ cows. If the reported differences in lipid composition of the endometrium are reflected in the histotroph, activity of PPARG in conceptus of NZ cows could be enhanced by the greater amount of polyunsaturated fatty acids and reduced amounts of monounsaturated fatty acids as hypothesized by Ribeiro (2018) and potentially contribute for the greater success in pregnancy establishment in NZ cows compared with NA cows in pasture-based systems.

One strategy widely used in commercial herds is crossbreeding of complementary pure breeds not only to obtain a desired phenotype, but also to reduce inbreeding and maximize heterosis. The crossbreeding between Holstein and Jersey cattle, for instance, is common and aims to combine the high milk volume yield of Holsteins with the high solids content in milk of Jerseys. In addition, improvements in reproduction have been reported with this crossbreeding strategy (Ribeiro *et al*., 2011; 2016c). Ribeiro *et al*. (2016c) compared conceptus elongation of Holstein (HC) and Holstein-conceptus development on day 15 and tended to have greater concentration of IFNT in the uterine flushings. Contrary to HC cows that had some ovoid conceptuses, CC had only elongated conceptuses. Moreover, CC pregnant cows had greater concentration of anandamide in the uterine flush and increased transcript expression of *PPARG* in the conceptus. Crossbred cows also had greater concentrations of circulating ovarian steroids before, around and after AI.

It is interesting to note that both studies investigating conceptus elongation in groups of cows with distinct genetics and fertility reported differences in lipid metabolism of the pregnant uterus, which reinforces the importance of lipid metabolism in conceptus development of dairy cows and supports the overall positive effects of dietary supplementation of fat in reproduction of dairy cows (Santos *et al*., 2008; Rodney *et al*., 2015). In addition, both studies reported differences in concentration of circulating steroids, in which the genetic group of higher fertility (New Zealand Holstein or North American Holstein-Jersey crossbred) had greater concentrations of circulating steroids compared with the genetic group of lower fertility (purebred North American Holsteins). Moreover, a study that compared Holstein cows with high and low estimated breeding values (EBV) for calving interval and similar EBVs for production traits found important differences in concentrations of circulating progesterone and IGF1, estrous behavior, energy metabolism and incidence of uterine diseases postpartum (Butler, 2014), suggesting that genetics could influence multiple physiological and cellular requirements for successful elongation of the preimplantation conceptus in lactating dairy cows.

## Conclusions

**Figure 5:**
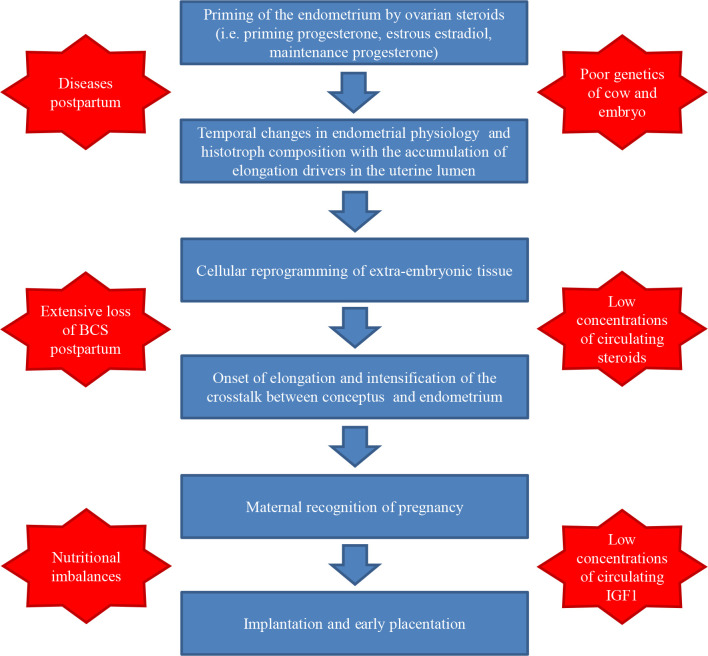
Summary of physiological and cellular requirements for elongation of the preimplantation bovine conceptus (represented in blue) and major factors affecting the success of elongation in lactating dairy cows (represented in red).

Successful elongation of the preimplantation conceptus depends on the modulation of endometrium physiology by steroid hormones, which include not only the effects of progesterone after ovulation but also the effects of estradiol during estrus and progesterone during the diestrus preceding estrus. The combined activity of priming hormones eventually leads to an environment that promotes the onset of conceptus elongation. Elongation requires reprogramming of cells in the extraembryonic tissues and involves changes in the cytoskeleton and adhesion molecules, increased in proliferation and migration of cells, and altered metabolism. Histotroph lipids are heavily utilized for synthesis of biomass, energy, production of prostaglandins, cell signaling, and regulation of gene expression though activity of the PPARG-RXR dimer of nuclear receptors. Abundance of PPARG-RXR ligands in the uterine histotroph is likely facilitated by the accumulation of lipid droplets and greater expression of RBP4 in the endometrium during diestrus. The highly active conceptus cells secrete bioactive factors that alter endometrium physiology and induce further changes in composition of the histotroph that are critical to attend the evolving demands of the expanding conceptus. Impairment of these physiological and cellular requirements could lead to failures in elongation and pregnancy losses (Fig. 5).

In lactating dairy cows, pregnancy losses are substantial and failures in the elongation process likely account for some of these losses. Lactation per se does not seem to impair elongation. However, elongation and survival of conceptuses seem to be affected in subgroups of lactating cows that present endocrine, metabolic and nutritional imbalances or deficiencies. These subgroups of cows include those that had health problems postpartum, anovular cows subjected to synchronization programs without supplementation of progesterone, cows with low concentrations of circulating IGF1, cows with reduced estrous behavior, and cows with reduced concentrations of progesterone after ovulation. In addition, elongation might be affected by genetics of cow and embryo, and by nutritional imbalances of protein, amino acids, minerals, vitamins, and fatty acids. Improving conceptus elongation and survival in lactating cows starts with optimization of health and nutrition programs during the transition period, includes the promotion of cow comfort and intake of complete and balanced diet, and can be extended to supplementation of specific deficiencies or stimulators such as essential fatty acids and somatotropin. Supplementation of steroids are mostly ineffective to promote survival of the conceptus except for progesterone supplementation during synchronization programs. Finally, the growing understanding of molecular regulation of reproduction is expected to result in the development of new fertility traits for genetic selection, which should emphasize pregnancy survival in order to consistently improve conception risk of dairy herds.

## References

[B1] Allrich RD. (1994). Endocrine and neural control of estrus in dairy cows. J Dairy Sci.

[B2] Baez GM, Barletta RV, Guenther JN, Gaska JM, Wiltbank MC. (2016). Effect of uterine size on fertility of lactating dairy cows. Theriogenology.

[B3] Barbosa L, Oliveira WVC, Pereira MHC, Moreira MB, Vasconcelos CGC, Silper BF, Cerri RLA, Vasconcelos JLM (2018). Somatic cell count and type of intramammary infection impacts fertility from in vitro produced embryo transfer. Theriogenology.

[B4] Barnwell CV, Farin PW, Ashwell CM, Farmer WT, Galphin SP, Farin CE (2016). Differences in mRNA populations of short and long bovine conceptuses on day 15 of gestation. Mol Reprod Dev.

[B5] Bauersachs S, Ulbrich SE, Gross K, Schmidt SE, Meyer HH, Einspanier R, Wenigerkind H, Vermehren M, Blum H, Sinowatz F, Wolf E. (2005). Gene expression profiling of bovine endometrium during the oestrous cycle: detection of molecular pathways involved in functional changes. J Mol Endocrinol.

[B6] Bauersachs S, Wolf E. (2012). Transcriptome analyses of bovine, porcine and equine endometrium during the pre-implantation phase. Anim Reprod Sci.

[B7] Bauersachs S, Simintiras CA, Sturmey RG, Krebs S, Bick J, Blum H, Wolf E, Lonergan P, Forde N. (2017). Effect of metabolic status on conceptus-maternal interactions on day 19 in dairy cattle: II. Effects on the endometrial transcriptome. Biol Reprod.

[B8] Bauman DE, Currie WB. (1980). Partitioning of nutrients during pregnancy and lactation: a review of mechanisms involving homeostasis and homeorhesis. J Dairy Sci.

[B9] Berger J, Moller DE. (2002). The mechanisms of action of PPARs. Annu Rev Med.

[B10] Betteridge KJ, Eaglesome MD, Randall GC, Mitchell D. (1980). Collection, description and transfer of embryos from cattle 10-16 days after oestrus. J Reprod Fertil.

[B11] Bindon BM. (1971). Role of progesterone in implantation in the sheep. Aust J Biol Sci.

[B12] Bisinotto RS, Chebel R, Santos JE. (2010). Follicular wave of the ovulatory follicle and not cyclic status influences fertility of dairy cows. J Dairy Sci.

[B13] Bisinotto RS, Ribeiro ES, Lima FS, Martinez N, Greco LF, Barbosa L, Bueno PP, Scagion LFS, Thatcher WW, Santos JEP. (2013). Targeted progesterone supplementation improves fertility in lactating dairy cows without a corpus luteum at the initiation of the timed artificial insemination protocol. J Dairy Sci.

[B14] Bisinotto RS, Lean IJ, Thatcher WW, Santos JE. (2015). Meta-analysis of progesterone supplementation during timed artificial insemination programs in dairy cows. J Dairy Sci.

[B15] Block J, Bonilla L, Hansen PJ (2010). Efficacy of in vitro embryo transfer in lactating dairy cows using fresh or vitrified embryos produced in a novel embryo culture medium. J Dairy Sci.

[B16] Boshier DP, Fairlough RJ, Holloway H (1987). Assessment of sheep blastocyst effects on neutral lipids in the uterine caruncular epithelium. J Reprod Fert.

[B17] Brinsfield TH, Hawk HW. (1973). Control by progesterone of the concentration of lipid droplets in epithelial cells of the sheep endometrium. J Anim Sci.

[B18] Brooks KE, Burns GW, Spencer TE. (2015). Peroxisome proliferator activator receptor gamma (PPARG) regulates conceptus elongation in sheep. Biol Reprod.

[B19] Butler WR. (2003). Energy balance relationships with follicular development, ovulation and fertility in postpartum dairy cows. Livest Prod Sci.

[B20] Butler ST, Marr AL, Pelton SH, Radcliff RP, Lucy MC, Butler WR. (2003). Insulin restores GH responsiveness during lactation-induced negative energy balance in dairy cattle: effects on expression of IGF-I and GH receptor 1A. J Endocrinol.

[B21] Butler ST. (2014). Genetic control of reproduction in dairy cows. Reprod Fertil Dev.

[B22] Cerri RL, Thompson IM, Kim IH, Ealy AD, Hansen PJ, Staples CR, Li JL, Santos JE, Thatcher WW. (2012). Effects of lactation and pregnancy on gene expression of endometrium of Holstein cows at day 17 of the estrous cycle or pregnancy. J Dairy Sci.

[B23] Carnegie JA, McCully ME, Robertson HA (1985). The early development of the sheep trophoblast and the involvement of cell death. Am J Anat.

[B24] Chagas LM, Bass JJ, Blache D, Burke CR, Kay JK, Lindsay DR, Lucy MC, Martin GB, Meier S, Rhodes FM, Roche JR, Thatcher WW, Webb R. (2007). Invited review: new perspectives on the roles of nutrition and metabolic priorities in the subfertility of high-producing dairy cows. J Dairy Sci.

[B25] Chebel RC, Demetrio DG, Metzger J (2008). Factors affecting success of embryo collection and transfer in large dairy herds. Theriogenology.

[B26] Clemente M, Lopez-Vidriero I, O’Gaora P, Mehta JP, Forde N, Gutierrez-Adan A, Lonergan P, Rizos D. (2011). Transcriptome changes at the initiation of elongation in the bovine conceptus. Biol Reprod.

[B27] Cole JB, Null DJ, VanRaden PM. (2016). Phenotypic and genetic effects of recessive haplotypes on yield, longevity, and fertility. J Dairy Sci.

[B28] Costello LM, O'Boyle P, Godkin JD, Diskin MG, Hynes AC, Morris DG. (2010). Retinol-binding protein (RBP), retinol and beta-carotene in the bovine uterus and plasma during the oestrous cycle and the relationship between systemic progesterone and RBP on day 7. Reprod Fertil Dev.

[B29] Drackley JK. (1999). ADSA Foundation Scholar Award. Biology of dairy cows during the transition period: the final frontier?. J Dairy Sci.

[B30] Ferraz PA, Burnley C, Karanja J, Viera-Neto A, Santos JE, Chebel RC, Galvao KN (2016). Factors affecting the success of a large embryo transfer program in Holstein cattle in a commercial herd in the southeast region of the United States. Theriogenology.

[B31] Foote WD, Gooch LD, Pope AL, Casida LE. (1957). The maintenance of early pregnancy in ovariectomised ewes by injections of ovarian hormones. J Anim Sci.

[B32] Forde N, Carter F, Fair T, Crowe MA, Evans AC, Spencer TE, Bazer FW, McBride R, Boland MP, O'Gaora P, Lonergan P, Roche JF. (2009). Progesterone-regulated changes in endometrial gene expression contribute to advanced conceptus development in cattle. Biol Reprod.

[B33] Forde N, Spencer TE, Bazer FW, Song G, Roche JF, Lonergan P. (2010). Effect of pregnancy and progesterone concentration on expression of genes encoding for transporters or secreted proteins in the bovine endometrium. Physiol Genomics.

[B34] Forde N, Beltman ME, Duffy GB, Duffy P, Mehta JP, O'Gaora P, Roche JF, Lonergan P, Crowe MA. (2011). Changes in the endometrial transcriptome during the bovine estrous cycle: effect of low circulating progesterone and consequences for conceptus elongation. Biol Reprod.

[B35] Forde N, McGettigan PA, Mehta JP, O'Hara L, Mamo S, Bazer FW, Spencer TE, Lonergan P. (2014). Proteomic analysis of uterine fluid during the pre- implantation period of pregnancy in cattle. Reproduction.

[B36] Forde N, Simintiras C A, Sturmey R G, Graf A, Wolf E, Blum H, Lonergan P. (2017). Effect of lactation on conceptus-maternal interactions at the initiation of implantation in cattle: I. Effects on the conceptus transcriptome and amino acid composition of the uterine luminal fluid. Biol Reprod.

[B37] França MR, da Silva MIS, Pugliesi G, Hoeck VV, Binelli M. (2017). Evidence of endometrial amino acid metabolism and transport modulation by peri-ovulatory endocrine profiles driving uterine receptivity. J Anim Sci Biotechnol.

[B38] Gao H, Wu G, Spencer TE, Johnson GA, Bazer FW. (2009). Select nutrients in the ovine uterine lumen. II. glucose transporters in the uterus and peri-implantation conceptuses. Biol Reprod.

[B39] Garrett JE, Geisert RD, Zavy MT, Morgan GL. (1988). Evidence for maternal regulation of early conceptus growth and development in beef cattle. Reproduction.

[B40] Geary TW, Burns GW, Moraes JG, Moss JI, Denicol AC, Dobbs KB, Ortega MS, Hansen PJ, Wehrman ME, Neibergs H, O'Neil E, Behura S, Spencer TE. (2016). Identification of beef heifers with superior uterine capacity for pregnancy. Biol Reprod.

[B41] Geisert RD, Morgan GL, Short EC, Zavy MT (1992). Endocrine events associated with endometrial function and conceptus development in cattle. Reprod Fertil Dev.

[B42] Goff JP. (2004). Macromineral disorders of the transition cow. Vet Clin North Am Food Anim Pract.

[B43] Groebner AE, Rubio-Aliaga I, Schulke K, Reichenbach HD, Daniel H, Wolf E, Meyer HH, Ulbrich SE. (2011). Increase of essential amino acids in the bovine uterine lumen during preimplantation development. Reproduction.

[B44] Han Y, Penagaricano F. (2016). Unravelling the genomic architecture of bull fertility in Holstein cattle. BMC Genet.

[B45] Hillegass J, Lima FS, Sa MF, Santos JE. (2008). Effect of time of artificial insemination and supplemental estradiol on reproduction of lactating dairy cows. J Dairy Sci.

[B46] Hue I, Degrelle SA, Campion E, Renard JP. (2007). Gene expression in elongating and gastrulating embryos from ruminants. Soc Reprod Fertil Suppl.

[B47] Hue I, Degrelle SA, Turenne N. (2012). Conceptus elongation in cattle: genes, models and questions. Anim Reprod Sci.

[B48] Hugentobler SA, Diskin MG, Leese HJ, Humpherson PG, Watson T, Sreenan JM, Morris DG. (2007). Amino acids in oviduct and uterine fluid and blood plasma during the estrous cycle in the bovine. Mol Reprod Dev.

[B49] Itoh T, Fairall L, Amin K, Inaba Y, Szanto A, Balint BL, Nagy L, Yamamoto K, Schwabe JW. (2008). Structural basis for the activation of PPARgamma by oxidized fatty acids. Nat Struct Mol Biol.

[B50] Kenyon AG, Lopes G, Mendonca LG, Lima JR, Bruno RG, Denicol AC, Chebel RC (2012). Ovarian responses and embryo survival in recipient lactating Holstein cows treated with equine chorionic gonadotropin. Theriogenology.

[B51] Kenyon AG, Mendonca LG, Lopes G, Lima JR, Santos JE, Chebel RC (2013). Minimal progesterone concentration required for embryo survival after embryo transfer in lactating Holstein cows. Anim Reprod Sci.

[B52] Kim J, Sato M, Li Q, Lydon JP, DeMayo FJ, Bagchi IC, Bagchi MK. (2008). Peroxisome proliferator- activated receptor γ is a target of progesterone regulation in the preovulatory follicles and controls ovulation in mice. Mol Cel Biol.

[B53] Kim J, Erikson DW, Burghardt RC, Spencer TE, Wu G, Bayless KJ, Johnson GA, Bazer FW. (2010). Secreted phosphoprotein 1 binds integrins to initiate multiple cell signaling pathways, including FRAP1/mTOR, to support attachment and force- generated migration of trophectoderm cells. Matrix Biol.

[B54] Kliewer SA, Sundseth SS, Jones SA, Brown PJ, Wisely GB, Koble CS, Devchand P, Wahli W, Willson TM, Lenhard JM, Lehmann JM. (1997). Fatty acids and eicosanoids regulate gene expression through direct interactions with peroxisome proliferator- activated receptors alpha and gamma. Proc Natl Acad Sci USA.

[B55] Le Roith D, Bondy C, Yakar S, Liu JL, Butler A (2001). The somatomedin hypothesis: 2001. Endocr Rev.

[B56] LeBlanc SJ, Herdt TH, Seymour WM, Duffield TF, Leslie KE. (2004). Peripartum serum vitamin E, retinol, and beta-carotene in dairy cattle and their associations with disease. J Dairy Sci.

[B57] Lonergan P, Forde N. (2014). Maternal-embryo interaction leading up to the initiation of implantation of pregnancy in cattle. Animal.

[B58] Madsen CA, Perry GA, Mogck CL, Daly RF, MacNeil MD, Geary TW. (2015). Effects of preovulatory estradiol on embryo survival and pregnancy establishment in beef cows. Anim Reprod Sci.

[B59] Maillo V, Rizos D, Besenfelder U, Havlicek V, Kelly AK, Garrett M, Lonergan P. (2012). Influence of lactation on metabolic characteristics and embryo development in postpartum Holstein dairy cows. J Dairy Sci.

[B60] Mamo S, Mehta JP, McGettigan P, Fair T, Spencer TE, Bazer FW, Lonergan P. (2011). RNA sequencing reveals novel gene clusters in bovine conceptuses associated with maternal recognition of pregnancy and implantation. Biol Reprod.

[B61] Mamo S, Mehta JP, Forde N, McGettigan P, Lonergan P. (2012). Conceptus-endometrium crosstalk during maternal recognition of pregnancy in cattle. Biol Reprod.

[B62] Marinho LS, Sanches BV, Rosa CO, Tannura JH, Rigo AG, Basso AC, Pontes JH, Seneda MM (2015). Pregnancy Rates to Fixed Embryo Transfer of Vitrified IVP Bos indicus, Bos taurus or Bos indicus x Bos taurus Embryos. Reprod Domest Anim.

[B63] Meier S, Peterson AJ, Mitchell MD, Littlejohn M, Walker CG, Roche JR. (2009). Genetic strain and reproductive status affect endometrial fatty acid concentrations. J Dairy Sci.

[B64] Mikkola M, Andersson M, Taponen J (2015). Transfer of cattle embryos produced with sex-sorted semen results in impaired pregnancy rate and increased male calf mortality. Theriogenology.

[B65] Miller BG, Moore NW. (1976). Effects of progesterone and oestradiol on endometrial metabolism and embryo survival in the ovariectomized ewe. Theriogenology.

[B66] Miller BG, Moore NW, Murphy L, Stone GM. (1977). Early pregnancy in the ewe: effects of oestradiol and progesterone on uterine metabolism and on embryo survival. Aust J Biol Sci.

[B67] Mitko K, Ulbrich SE, Wenigerkind H, Sinowatz F, Blum H, Wolf E, Bauersachs S. (2008). Dynamic changes in messenger RNA profiles of bovine endometrium during the oestrous cycle. Reproduction.

[B68] Monteiro PL, Ribeiro ES, Maciel RP, Dias AL, Sole E, Lima FS, Bisinotto RS, Thatcher WW, Sartori R, Santos JE (2014). Effects of supplemental progesterone after artificial insemination on expression of interferon-stimulated genes and fertility in dairy cows. J Dairy Sci.

[B69] Monteiro PL, Nascimento AB, Pontes GCS, Fernandes GO, Melo LF, Wiltbank MC, Sartori R. (2015). Progesterone supplementation after ovulation: Effects on corpus luteum function and on fertility of dairy cows subjected to AI or ET. Theriogenology.

[B70] Moore NW, Rowson LE. (1959). Maintenance of pregnancy in ovariectomized ewes by means of progesterone. Nature.

[B71] Moore NW, Shelton JN. (1964). Egg transfer in sheep. effect of degree of synchronization between donor and recipient, age of egg, and site of transfer on the survival of transferred eggs. J Reprod Fertil.

[B72] Moore NM. (1985). The use of embryo transfer and steroid hormone replacementtherapy in the study of prenatal mortality. Theriogenology.

[B73] Moraes JGN, Behura SK, Geary TW, Hansen PJ, Neibergs HL, Spencer TE. (2018). Uterine influences on conceptus development in fertility-classified animals. Proc Natl Acad Sci USA.

[B74] Mullen MP, Elia G, Hilliard M, Parr MH, Diskin MG, Evans AC, Crowe MA. (2012a). Proteomic characterization of histotroph during the preimplantation phase of the estrous cycle in cattle. J Proteome Res.

[B75] Mullen MP, Forde N, Parr MH, Diskin MG, Morris DG, Nally JE, Evans AC, Crowe MA. (2012b). Alterations in systemic concentrations of progesterone during the early luteal phase affect RBP4 expression in the bovine uterus. Reprod Fertil Dev.

[B76] Nagy L, Tontonoz P, Alvarez JGA, Chen H, Evans RM. (1998). Oxidized LDL regulates macrophage gene expression through ligand activation of PPARγ. Cell.

[B77] Okumu LA, Forde N, Fahey AG, Fitzpatrick E, Roche JF, Crowe MA, Lonergan P. (2010). The effect of elevated progesterone and pregnancy status on mRNA expression and localisation of progesterone and oestrogen receptors in the bovine uterus. Reproduction.

[B78] Ortega MS, Denicol AC, Cole JB, Null DJ, Hansen PJ. (2016). Use of single nucleotide polymorphisms in candidate genes associated with daughter pregnancy rate for prediction of genetic merit for reproduction in Holstein cows. Anim Genet.

[B79] Parr MH, Crowe MA, Lonergan P, Evans AC, Rizos D, Diskin MG. (2014). Effect of exogenous progesterone supplementation in the early luteal phase post- insemination on pregnancy per artificial insemination in Holstein-Friesian cows. Anim Reprod Sci.

[B80] Pereira MH, Sanches CP, Guida TG, Rodrigues AD, Aragon FL, Veras MB, Borges PT, Wiltbank MC, Vasconcelos JL (2013). Timing of prostaglandin F2alpha treatment in an estrogen-based protocol for timed artificial insemination or timed embryo transfer in lactating dairy cows. J Dairy Sci.

[B81] Pereira MHC, Wiltbank MC, Vasconcelos JLM. (2016). Expression of estrus improves fertility and decreases pregnancy losses in lactating dairy cows that receive artificial insemination or embryo transfer. J Dairy Sci.

[B82] Pereira MHC, Wiltbank MC, Guida TG, Lopes FR, Vasconcelos JLM (2017). Comparison of 2 protocols to increase circulating progesterone concentration before timed artificial insemination in lactating dairy cows with or without elevated body temperature. J Dairy Sci.

[B83] Pinaffi FLV, Araujo ER, Silva LA, Ginther OJ. (2017). Color-Doppler signals of blood flow in the corpus luteum and vascular perfusion index for ovarian and uterine arteries during expansion of the allantochorion in Bos taurus heifers. Theriogenology.

[B84] Reese ST, Pereira MHC, Edwards JL, Vasconcelos JLM, Pohler KG (2018). Pregnancy diagnosis in cattle using pregnancy associated glycoprotein concentration in circulation at day 24 of gestation. Theriogenology.

[B85] Rhoads ML, Meyer JP, Kolath SJ, Lamberson WR, Lucy MC. (2008). Growth hormone receptor, insulin-like growth factor (IGF)-1, and IGF-binding protein-2 expression in the reproductive tissues of early postpartum dairy cows. J Dairy Sci.

[B86] Rhodes FM, McDougall S, Burke CR, Verkerk GA, Macmillan KL. (2003). Treatment of cows with an extended postpartum anestrous interval. J. Dairy Sci.

[B87] Ribeiro ES, Cerri RL, Bisinotto RS, Lima FS, Silvestre FT, Greco LF, Thatcher WW, Santos JE. (2011). Reproductive performance of grazing dairy cows following presynchronization and resynchronization protocols. J Dairy Sci.

[B88] Ribeiro ES, Galvão K, Thatcher WW, Santos JEP. (2012). Economic aspects of applying reproductive technologies to dairy herds. Anim Reprod.

[B89] Ribeiro ES, Lima FS, Greco LF, Bisinotto RS, Monteiro AP, Favoreto M, Ayres H, Marsola RS, Martinez N, Thatcher WW, Santos JE. (2013). Prevalence of periparturient diseases and effects on fertility of seasonally calving grazing dairy cows supplemented with concentrates. J Dairy Sci.

[B90] Ribeiro ES, Bisinotto RS, Lima FS, Greco LF, Morrison A, Kumar A, Thatcher WW, Santos JE. (2014a). Plasma anti-Mullerian hormone in adult dairy cows and associations with fertility. J Dairy Sci.

[B91] Ribeiro ES, Bruno RG, Farias AM, Hernandez- Rivera JA, Gomes GC, Surjus R, Becker LF, Birt A, Ott TL, Branen JR, Sasser RG, Keisler DH, Thatcher WW, Bilby TR, Santos JE. (2014b). Low doses of bovine somatotropin enhance conceptus development and fertility in lactating dairy cows. Biol Reprod.

[B92] Ribeiro ES, Greco LF, Bisinotto RS, Lima FS, Thatcher WW, Santos JE. (2016a). Biology of preimplantation conceptus at the onset of elongation in dairy cows. Biol Reprod.

[B93] Ribeiro ES, Gomes GC, Greco LF, Cerri RLA, Vieira-Neto A, Monteiro PLJ, Lima FS, Bisinotto RS, Thatcher WW, Santos JEP (2016b). Carryover effect of postpartum inflammatory diseases on developmental biology and fertility in lactating dairy cows. J Dairy Sci.

[B94] Ribeiro ES, Monteiro APA, Bisinotto RS, Lima FS, Greco LF, Ealy AD, Thatcher WW, Santos JEP. (2016c). Conceptus development and transcriptome at preimplantation stages in lactating dairy cows of distinct genetic groups and estrous cyclic statuses. J Dairy Sci.

[B95] Ribeiro ES, Santos JE, Thatcher WW. (2016d). Role of lipids on elongation of the preimplantation conceptus in ruminants. Reproduction.

[B96] Ribeiro ES, Carvalho MR. (2017). Impact and mechanisms of inflammatory diseases on embryonic development and fertility in cattle. Animal Reproduction.

[B97] Ribeiro ES. (2018). Lipids as regulators of conceptus development:Implications for metabolic regulation of reproduction in dairy cattle. J Dairy Sci.

[B98] Rivera FA, Mendonca LG, Lopes G, Santos JE, Perez RV, Amstalden M, Correa-Calderon A, Chebel RC (2011). Reduced progesterone concentration during growth of the first follicular wave affects embryo quality but has no effect on embryo survival post transfer in lactating dairy cows. Reproduction.

[B99] Robinson RS, Mann GE, Gadd TS, Lamming GE, Wathes DC. (2000). The expression of the IGF system in the bovine uterus throughout the oestrous cycle and early pregnancy. J Endocrinol.

[B100] Rodney RM, Celi P, Scott W, Breinhild K, Lean IJ. (2015). Effects of dietary fat on fertility of dairy cattle: a meta-analysis and meta-regression. J Dairy Sci.

[B101] Romero JJ, Liebig BE, Broeckling CD, Prenni JE, Hansen TR. (2017). Pregnancy-induced changes in metabolome and proteome in ovine uterine flushings. Biol Reprod.

[B102] Santos JE, Thatcher WW, Chebel RC, Cerri RL, Galvao KN. (2004). The effect of embryonic death rates in cattle on the efficacy of estrus synchronization programs. Anim Reprod Sci.

[B103] Santos JE, Bilby TR, Thatcher WW, Staples CR, Silvestre FT. (2008). Long chain fatty acids of diet as factors influencing reproduction in cattle. Reprod Domest Anim.

[B104] Santos JE, Rutigliano HM, Sá MF. (2009). Risk factors for resumption of postpartum estrous cycles and embryonic survival in lactating dairy cows. Anim Reprod Sci.

[B105] Santos JE, Bisinotto RS, Ribeiro ES, Lima FS, Greco LF, Staples CR, Thatcher WW. (2010). Applying nutrition and physiology to improve reproduction in dairy cattle. Soc Reprod Fertil Suppl.

[B106] Santos JE, Bisinotto RS, Ribeiro ES. (2016). Mechanisms underlying reduced fertility in anovular dairy cows. Theriogenology.

[B107] Sawai K, Kageyama S, Moriyasu S, Hirayama H, Minamihashi A, Onoe S. (2007). Changes in the mRNA transcripts of insulin-like growth factor ligand, receptors and binding proteins in bovine blastocysts and elongated embryos derived from somatic cell nuclear transfer. J Reprod Dev.

[B108] Schinckel PG. (1954). The effect of the presence of the ram on the ovarian activity of the ewe. Aust J Agric Res.

[B109] Sellars CB, Dalton JC, Manzo R, Day J, Ahmadzadeh A. (2006). Time and incidence of ovulation and conception rates after incorporating estradiol cypionate into a timed artificial insemination protocol. J Dairy Sci.

[B110] Shimizu T, Krebs S, Bauersachs S, Blum H, Wolf E, Miyamoto A. (2010). Actions and interactions of progesterone and estrogen on transcriptome profiles of the bovine endometrium. Physiol Genomics.

[B111] Simmons RM, Erikson DW, Kim J, Burghardt RC, Bazer FW, Johnson GA, Spencer TE. (2009). Insulin- like growth factor binding protein-1 in the ruminant uterus: potential endometrial marker and regulator of conceptus elongation. Endocrinology.

[B112] Souza AH, Gumen A, Silva EP, Cunha AP, Guenther JN, Peto CM, Caraviello DZ, Wiltbank MC. (2007). Supplementation with estradiol-17beta before the last gonadotropin-releasing hormone injection of the Ovsynch protocol in lactating dairy cows. J Dairy Sci.

[B113] Spencer TE, Bazer FW. (1995). Temporal and spatial alterations in uterine estrogen receptor and progesterone receptor gene expression during the estrous cycle and early pregnancy in the ewe. Biol Reprod.

[B114] Spencer TE, Johnson GA, Burghardt RC, Bazer FW. (2004). Progesterone and placental hormone actions on the uterus: insights from domestic animals. Biol Reprod.

[B115] Spencer TE, Forde N, Dorniak P, Hansen TR, Romero JJ, Lonergan P. (2013). Conceptus-derived prostaglandins regulate gene expression in the endometrium prior to pregnancy recognition in ruminants. Reproduction.

[B116] Sponchiado M, Gomes NS, Fontes PK, Martins T, Del Collado M, Pastore AA, Pugliesi G, Nogueira MFG, Binelli M. (2017). Pre-hatching embryo-dependent and -independent programming of endometrial function in cattle. PLoS One.

[B117] Stewart BM, Block J, Morelli P, Navarette AE, Amstalden M, Bonilla L, Hansen PJ, Bilby TR (2011). Efficacy of embryo transfer in lactating dairy cows during summer using fresh or vitrified embryos produced in vitro with sex-sorted semen. J Dairy Sci.

[B118] Thatcher WW, Macmillan KL, Hansen PJ, Drost M. (1989). Concepts for regulation of corpus luteum function by the conceptus and ovarian follicles to improve fertility. Theriogenology.

[B119] Thatcher WW, Moreira F, Santos JE, Mattos RC, Lopes FL, Pancarci SM, Risco CA (2001). Effects of hormonal treatments on reproductive performance and embryo production. Theriogenology.

[B120] Thiam AR, Farese RV, Walther TC (2013). The biophysics and cell biology of lipid droplets. Nat Rev Mol Cell Biol.

[B121] Thompson IM, Cerri RL, Kim IH, Ealy AD, Hansen PJ, Staples CR, Thatcher WW. (2012). Effects of lactation and pregnancy on metabolic and hormonal responses and expression of selected conceptus and endometrial genes of Holstein dairy cattle. J Dairy Sci.

[B122] Ulbrich SE, Schulke K, Groebner AE, Reichenbach HD, Angioni C, Geisslinger G, Meyer HH. (2009). Quantitative characterization of prostaglandins in the uterus of early pregnant cattle. Reproduction.

[B123] Valour D, Degrelle SA, Ponter AA, Giraud-Delville C, Campion E, Guyader-Joly C, Richard C, Constant F, Humblot P, Ponsart C, Hue I, Grimard B. (2014). Energy and lipid metabolism gene expression of D18 embryos in dairy cows is related to dam physiological status. Physiol Genomics.

[B124] Wales RG, Cuneo CL. (1989). Morphology and chemical analysis of the sheep conceptus from the 13th to the 19th day of pregnancy. Reprod Fertil Dev.

[B125] Wang J, Guillomot M, Hue I. (2009). Cellular organization of the trophoblastic epithelium in elongating conceptuses of ruminants. C R Biol.

[B126] Wiltbank MC, Gumen A, Sartori R. (2002). Physiological classification of anovulatory conditions in cattle. Theriogenology.

[B127] Wiltbank MC, Lopez H, Sartori R, Sangsritavong S, Gumen A. (2006). Changes in reproductive physiology of lactating dairy cows due to elevated steroid metabolism. Theriogenology.

[B128] Wiltbank MC, Souza AH, Carvalho PD, Cunha AP, Giordano JO, Fricke PM, Baez GM, Diskin MG. (2014). Physiological and practical effects of progesterone on reproduction in dairy cattle. Animal.

[B129] Wiltbank MC, Baez GM, Garcia-Guerra A, Toledo MZ, Monteiro PL, Melo LF, Ochoa JC, Santos JE, Sartori R. (2016). Pivotal periods for pregnancy loss during the first trimester of gestation in lactating dairy cows. Theriogenology.

[B130] Wordinger RJ, Dickey JF, Ellicott AR. (1977). Histochemical evaluation of the lipid droplet content of bovine oviductal and endometrial epithelial cells. J Reprod Fertil.

[B131] Yang X, Zhang W, Chen Y, Li Y, Sun L, Liu Y, Liu M, Yu M, Li X, Han J, Duan Y. (2016). Activation of peroxisome proliferator-activated receptor γ (PPARγ) and CD36 protein expression. J Biol Chem.

[B132] Yaseen MA, Wrenzycki C, Herrmann D, Carnwath JW, Niemann H. (2001). Changes in the relative abundance of mRNA transcripts for insulin-like growth factor (IGF-I and IGF-II) ligands and their receptors (IGF-IR/IGF-IIR) in preimplantation bovine embryos derived from different in vitro systems. Reproduction.

